# Nanomedicine for the poor: a lost cause or an idea whose time has yet to come?

**DOI:** 10.2217/nnm-2021-0024

**Published:** 2021-05-14

**Authors:** Vuk Uskoković

**Affiliations:** ^1^Advanced Materials & Nanobiotechnology Laboratory, TardigradeNano LLC, Irvine, CA 92604, USA

**Keywords:** COVID-19, Gini coefficient, intellectual property, nanotechnology, QALY, SARS-CoV-2, social equality, vaccines

## Abstract

The most effective COVID-19 vaccines, to date, utilize nanotechnology to deliver immunostimulatory mRNA. However, their high cost equates to low affordability. Total nano-vaccine purchases per capita and their proportion within the total vaccine lots have increased directly with the GDP per capita of countries. While three out of four COVID-19 vaccines procured by wealthy countries by the end of 2020 were nano-vaccines, this amounted to only one in ten for middle-income countries and nil for the low-income countries. Meanwhile, economic gains of saving lives with nano-vaccines in USA translate to large costs in middle-/low-income countries. It is discussed how nanomedicine can contribute to shrinking this gap between rich and poor instead of becoming an exquisite technology for the privileged. Two basic routes are outlined: (1) the use of qualitative contextual analyses to endorse R&D that positively affects the sociocultural climate; (2) challenging the commercial, competitive realities wherein scientific innovation of the day operates.

## Tweetable abstract

How could nanomedicine heal the social inequality divide? Discussed in the light of the race for the COVID-19 vaccine.

## Nanomedicine versus social inequalities: bottom-up perspective

Immoderate inequalities within a society are one of the most reliable predictors of the social welfare, scaling reciprocally with it. Moreover, research has shown that an increase in the Gini coefficient, a measure of the income inequality, directly leads to social inequity in unmet healthcare needs [1,2]. The Gini coefficient, for example, has been shown to correlate directly with the mortalities due to COVID-19 across USA [3], as well as with the number of reported cases of malaria across different regions of Africa [4]. In other words, the growing gap between those who have and those who have not has severe repercussions on medical care.

The question to be raised here is where research at the frontier of biomedicine, including nanomedicine, lies within this grave trend. How is the innovation in this field of science affected by these economic considerations and, in turn, how does this research affect these inequalities? We, as scientists, prefer to remain undefiled by the harsh hand of politics; however, science is a social endeavor. It is an exercise in interpersonal communication [5] and it is inextricably tangled with socioeconomic issues. Any scientists, particularly if out of work or resources for research, are aware of how politics and economy implacably affect science. However, the ways in which the choice of topics for scientific research affects the sociocultural climate are far more sensitive and difficult to grasp.

Technologies are often assumed to be neutral. But just as no experimental observations are impartial because in every question lurks a premise resting on a bed of beliefs, an answer as it were, there are no neutral technologies either. Each technology, as Heidegger had it, predisposes its user for a specific way of being [6]. One example from the realm of fine arts, albeit readily translatable to the scientific domain, may suffice here. It is of the two predominant materials used by Renaissance sculptors: the terracotta clay and marble. Whereas the more moldable character of the former material enabled the artists, such as Torrigiano and dell'Arca, to create more realistic expressions of the sculpted figures, the more lustrous and exquisite character of the latter material made art made out of it, as in the works by Michelangelo and Donatello, more transcendent in nature.

Innovations in medical technology have, likewise, had their say in shaping human values and modes of communication. Take biomaterials, for example: their extensive application leads to the dream about a bionic man, which may bring with it more computerized, if not purely perfunctory modes of communication. The lifesaving quality of prosthetics notwithstanding, many of the dystopian sci-fi auteurs have elaborated on this Promethean adoption of inhumane traits by human characters who have been turned into cyborgs [7,8]. Nanomedicine *per se*, however, is a step up on the ladder of technical finesse compared with medicine alone. From its inception to this day, it has connoted the use of the most advanced technological tools for medical ends, for which reason discussions over its socioeconomic effects are of critical importance, even though they are thoroughly missing from today's discourse. Still, some examples of these effects can be proposed here. For one, a medical nanotechnology such as nanoparticles as contrast agents in radiographic imaging promotes clinical diagnostic modalities and a specific infrastructure and workforce tied with them. It also implicitly promotes the representation of the patient in geometric terms rather than as entangled pathways of biochemical reactions, which diagnostic chemical assays would naturally lead to. In contrast, a nanotechnology such as nanoparticles for targeted drug delivery facilitates a flexible and more decentralized network of medical points of care, gravitating more toward pharmacy practice as opposed to the reliance on massive medical instrumentation. Yet another example can be that of nanoparticles usable in genomic sequencing and gene expression analyses such as qPCR, which might lead to the reaffirmation of the premises of personalized medicine, along with its specific, but also isolationistic connotations. Personalized medicine, we know, can create obstacles for drug development, similar to that associated with the rare diseases, which large biotech companies find less financially rewarding to invest in compared with universal drugs, be they called aspirin, remdesivir, remeron or something else. Therefore, it becomes apparent how the birth and the fosterage of a new technology can change the socioeconomic aspects of the social system in which it is applied, notwithstanding the complexity of the network of causes and effects surrounding it, severely oversimplified in the aforementioned examples.

So how can science and technology, including that pertaining to the field of nanomedicine, contribute to the narrowing of the detrimental gap between the rich and the poor? The starting point should be the awakening of the awareness of their influence on social equality, the deepening of which through comprehensive qualitative and quantitative analyses may lead to choices empowered by rational arguments. On one hand, being more accessible to the poor, inexpensive technologies are a natural healer of this notorious gap. However, it would be too simplistic to assume that minimization of the costs of resources, of production methods, of storage and administration are the only criteria needed to be satisfied to make technologies universally affordable. This is because this would only devalue the hi-tech philosophy which developed countries have worked hard to have the liberty to pursue. It would also be an extension of the materialistic economic paradigm that all the poor need is to increase their wealth [9], which is a view that can be valid only if and when it gets complemented with analytical perspectives that take into account the cultural, ecological, humanistic and, why not, spiritual corollaries of the economic growth. Therefore, the overall zeitgeist and the broad contexts of application should be considered before bringing about the decision as to what constitutes a scientific method in favor of social equity and what is an approach with an adverse effect on it. Advocating nanomaterials such as nanocellulose, carbon dots or calcium phosphate (CaP) nanoparticles as medical materials for healing the social inequality gap only because they are comparatively inexpensive and impose a negligible ecological footprint is thus insufficient and needs to be supplemented with other types of inquiry. The use of CaP, for example, as a means for bridging the gap between the rich and the poor has aesthetic connotations that may resonate with cultures other than the western and thus go beyond sheer economic considerations. The idiosyncrasy of CaP among similar bioceramics [10], its biogenic proliferation despite the intrinsic structural weaknesses [11], and its chemical resemblance to the skeletal foundations of our bodies [12] can carry such aesthetic connotations. CaP is also a material whose popularity is evenly distributed across the globe, meaning that the sole act of engaging in its research may be sufficient to bring people from various cultures together. This is in contrast with the more exclusionary technologies, which leave the researchers with limited resources behind and contribute to widening the gap of inequality [13]. In general, small, adaptable technologies that resonate with the local cultures and are more smoothly implementable by them are particularly desirable here in comparison with the hefty medical tools, not only because of the infrastructure compatibility issues and the costs of production, installation, training and/or maintenance, but also because today's rapid replacements on the pedestal of state-of-the-art instrumentation lead to incertitude about the duration of their currency. Consequently, rapid successions on the conveyer belt of innovation in the developed world are double-edged swords: they may bring about certain benefits, but are also prone to only further aggravate the existing gap between the rich and the poor.

## Example of COVID-19 nano-vaccines

The width of the inequality gap has a destabilizing social influence, making the system vulnerable under a slightest stress. This has become obvious in these days when the world is gripped by a deadly pandemic. The inability to curb the spread of infection regardless of the measures in place has insinuated profuse connections between people, even when these connections seem nonexistent. It has reminded the developed world that the burden of infectious diseases that plagues the daily life of many poor countries in the form of malaria or tuberculosis is only a whisker away [14]. It has also demonstrated how connected various aspects of a society are, as by affecting people's health, SARS-CoV-2 has put pressure not only on the systems of healthcare, but on everything else too, from limiting opportunities for scientific and other work to posing challenges for interpersonal communication to giving rise to various political schisms. Logically, in the geopolitically divided world of 2020, too focused on short-term interests to be able to consider the ecosphere as a network where everything is, more or less, connected to everything else, a concerted global commitment needed to effectively resolve a communicable disease pandemic such as COVID-19 has been thoroughly lacking.

Correspondingly, deliberation over the case of vaccines for COVID-19 can be instructive here by shedding more light on this relationship between nanotechnologies and social equity. On one hand, the fact that the vaccines BNT162b1 (*Pfizer-BioNTech*) and mRNA-1273 (*Moderna*) are the first to be approved and administered both in the countries of their origin and globally may act as a beacon of hope for both the socially aware scientist looking after closing this gap and the general scientist in the nanomedical field, considering that out of nine COVID-19 vaccines approved by 19 January 2021 and many more being on the way, these two are the only nano-vaccines, utilizing phospholipid nanoparticles as adjuvants and as delivery vehicles for immunostimulatory mRNA [15]. Nanoparticles, of course, have had a long history of use as adjuvants [16], even though traditional vaccines usually resort to simpler materials as boosters of the immune recognition of the antigens, such as alum, as in traditional tetanus and hepatitis B vaccines, inulin, as in some flu vaccines, or monophosphoryl lipid A, as in *Cervarix*. However, for the negatively charged and rather unstable mRNA, highly susceptible to proteolysis, to be endocytosed and translated into an antigenic protein, the right form of delivery is crucial, and this is where nanoparticles come to play as essential ingredients of these two vaccine formulations [17]. Therefore, the high price of these two mRNA vaccines is partly due to the use of mRNA encoding for the receptor-binding domain of the SARS-CoV-2 spike protein as the immunogen and partly due to the use of a nanoparticle carrier, but a significant contributor to the production cost also comes in the form of the nanotechnology integrating both of these ingredients into a drug delivery platform capable of achieving an effective intracellular transfer and transfection. Therefore, it should not surprise that the bottleneck in the mass production of mRNA vaccines for COVID-19, which significantly hampered the early immunization campaign, has come from the limited capacity to produce the lipid nanoparticles as carriers for the antigenic ribonucleic acids.

A major positive aspect of these novel mRNA vaccines is the relative ease with which the sequence of nucleotides in the immunogenic molecule could be altered using a simple oligo synthesizer, as opposed to the more laborious process of changing the amino acid sequence in protein vaccines, making the former potentially more effective against a pandemic where the viral agent rapidly creates new variants through mutation, as it is the case with SARS-CoV-2 [18]. It is too early to know whether this instant sequence building approach utilizing critical but short genetic codes will fare well against the rapidly mutating SARS-CoV-2 virus or the duration of the protection would be mediocre compared with the more traditional exposure to inactivated whole virus. In a worst case scenario, a constant reproduction of new vaccines would be necessitated to keep up with the new emerging virus variants and although this approach might benefit the vaccine producer, it may not necessarily benefit the people. Irrespective of these uncertainties, the method is unequivocally commendable for the rapid antigenic design that it facilitates. Its success serves as an implicit call for continued research on advanced nanotechnologies for controlled and targeted drug delivery, which is, of course, badly needed in a world where most local infections are treated with systemic antibiotics, a method as preposterous as the hypothetical treating of a malarial mosquito outbreak in the Mudzi region of Zimbabwe by deploying pesticide-spraying airplanes over every corner of the planet and having people in Patagonian prairies and Siberian steppes cough up the toxins. In fact, the better protection against the SARS-CoV-2 infection achieved by BNT162b1 and mRNA-1273 than by more traditional vaccines may be expected to expand the interest in nano-vaccines, and one example may be a recently reported vaccine candidate in the form of ferritin nanoparticles functionalized with shortened SARS-CoV-2 spike protein sequences [19]. Other types of nanoparticles studied with success for the delivery of mRNA in vaccines are those of polysaccharides, cholesterol, cationic dendrimers and other polymers [20], and their translation to the clinical testing stages and/or market should be expected soon.

**Figure 1.**
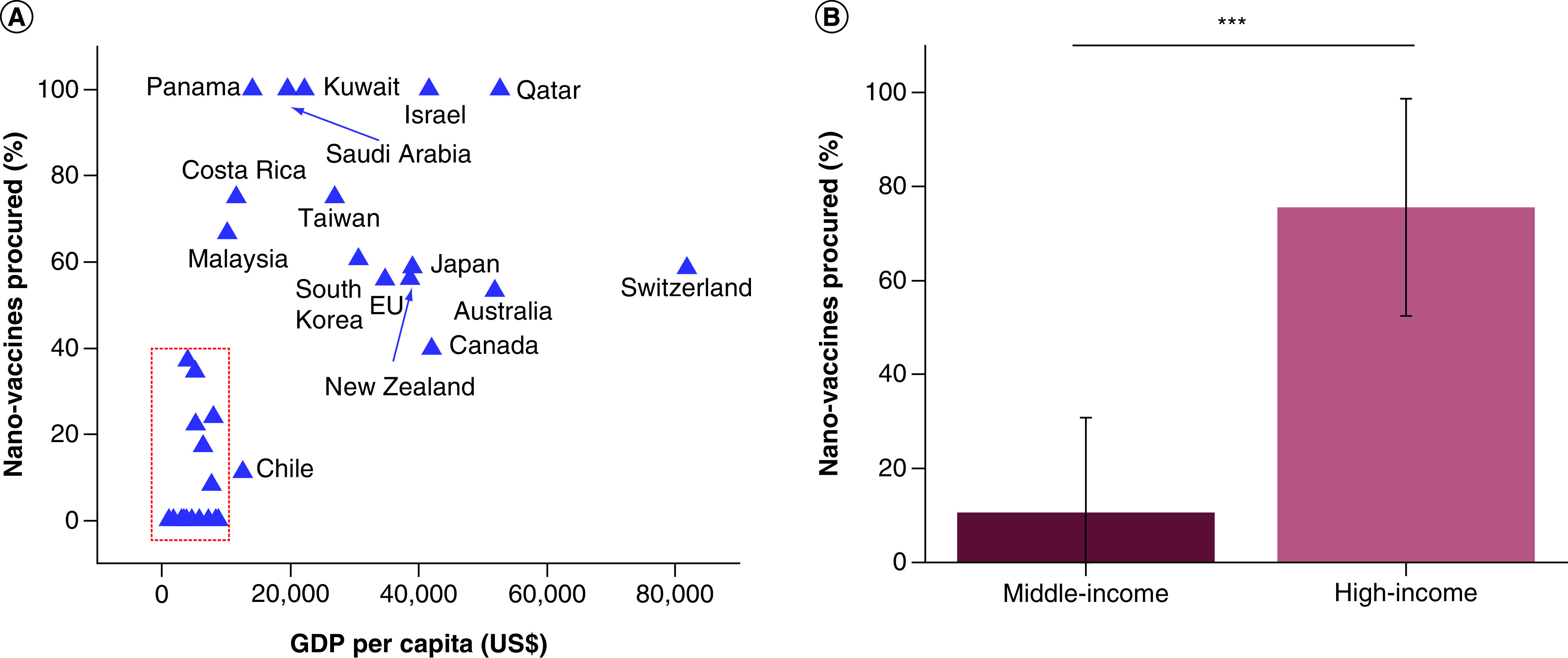
COVID-19 nano-vaccine procurement relative to total COVID-19 vaccine procurement. Percentage of COVID-19 nano-vaccines purchased by countries as a function of their nominal GDP per capita, as reported by the International Monetary Fund for 2020 **(A).** Among nano-vaccines were included one protein nanoparticle vaccine (NVX-CoV2373 [Novavax]) and four mRNA vaccines either in the approval stage or in advanced clinical trials at the time at which the statistical data were collected (11 December 2020): BNT162b1 (Pfizer-BioNTech), mRNA-1273 (Moderna), CVnCoV (CureVac) and ARCT-021 (Arcturus). Other vaccines included more traditional DNA (GX-19 [Genexine], INO-4800 (INOVIO)), non-replicating (AZD1222 [AstraZeneca], Ad26.COV2.S [Janssen], Gam-COVID-Vac [Gamaleya]), or replicating (TMV-083 [Merck]) viral vector based, inactivated (BBIBP-CorV [Sinopharm], CoronaVac [Sinovac], VLA2001 [Valneva]) or protein subunit (Sanofi*-*GSK) ones [25]. Most data points were collected from the Duke Global Health Innovation Center COVID-19 Launch and Scale Speedometer. Some data points were corrected by the author, e.g., Switzerland and Serbia, and some were added, e.g., Qatar. Countries with domestically produced vaccines, including China, Russia, UK and USA, have not been included in the analysis for bias concerns. Average percentage of COVID-19 nano-vaccines purchased by middle-income countries (US$1000 <GDP per capita <US$13,000) and high-income countries (>US$13,000 GDP per capita) by the end of 2020 relative to the total number of vaccines in their procured stocks **(B).** The total COVID-19 vaccine purchases by the poor countries (GDP per capita <US$1,000) were negligible by the same date and did not make it to the statistic. To estimate the boundaries between the high- and the middle-income countries and between the middle- and the low-income countries, nominal GDP was approximated as equivalent to the nominal gross national income, as for most countries these two values lie within a same range, differing by ∼ ± $500–2,000. The dashed rectangle denotes 32 unlabeled middle-income countries included in the analysis (India, Thailand, Mexico, Brazil, Argentina, Egypt, Morocco, etc), adding up to the total of 35 together with Chile, Costa Rica and Malaysia. The bars represent averages of n = 13 for the high-income countries including EU and n = 36 for the middle-income countries. Error bars represent standard deviation and *** represents an extremely statistically significant difference (p < 0.0001). GDP: Gross domestic product.

**Figure 2.**
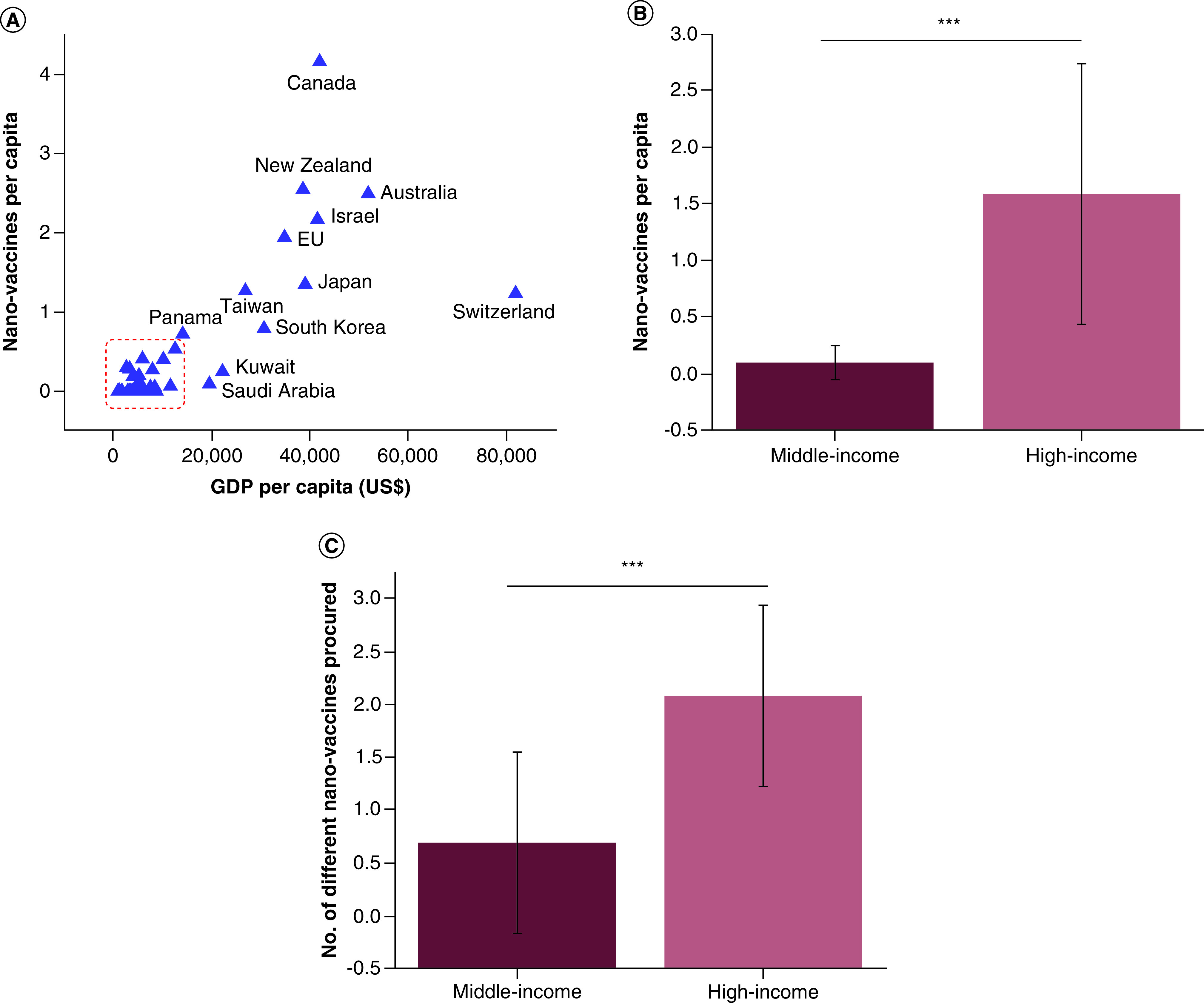
COVID-19 nano-vaccine procurement per capita and per type. Number of COVID-19 nano-vaccines per capita purchased by countries as a function of their nominal GDP per capita, as reported by the International Monetary Fund for 2020 **(A).** Among nano-vaccines were included one protein nanoparticle vaccine (NVX-CoV2373 (Novavax)) and four mRNA vaccines either approved for human use or in advanced clinical trials at the time at which the statistical data were collected (19 January 2021): BNT162b1 (Pfizer-BioNTech), mRNA-1273 (Moderna), CVnCoV (CureVac) and ARCT-021 (Arcturus). Data points were collected from the Duke Global Health Innovation Center COVID-19 Launch and Scale Speedometer. Countries with domestically produced vaccines, including China, Russia, UK and USA, have not been included in the analysis for bias concerns. Average number of COVID-19 nano-vaccines per capita purchased by middle-income countries (US$1,000 <GDP per capita <US$13,000) and high-income countries (>US$13,000 GDP per capita) by 19 January 2021 **(B).** Average number of different types of COVID-19 nano-vaccines purchased by middle-income countries and high-income countries by 19 January, 2021 **(C).** The dashed rectangle in **(A)** denotes 34 unlabeled middle-income countries included in the analysis. The bars in **(B)** and **(C)** represent averages of n = 12 for the high-income countries including EU and n = 34 for the middle-income countries. Error bars represent standard deviation and *** represents an extremely statistically significant difference (p < 0.0001). GDP: Gross domestic product.

On the other hand, however, it is gradually becoming obvious that the accessibility to these nano-vaccines is largely determined by the economic status of a country. For example, despite the fact that *Pfizer* and *Moderna* were the first to release their comparatively pricey mRNA nano-vaccines at $20 and $33 per dose (without including the cost of storage at temperatures lower than –20°C and –60°C, respectively), respectively, countries such as El Salvador, Thailand, Philippines, Vietnam, Bangladesh and the Dominican Republic procured by the end of 2020 only the cheapest of all vaccines in production, namely the chimpanzee adenovirus vectored vaccine ChAdOx1 nCoV-19 (AZD1222, *Oxford-AstraZeneca*), whose cost is $4 per dose [21]. A similar cost disparity can be noted for the first commercial nanomedicine, namely the PEGylated liposomal formulation of doxorubicin known as *Doxil*. With its cost of $873 for a 20 mg vial or, paradoxically, $888 for the generic version, it is considerably less available to patients on the budget compared with pure doxorubicin at the cost of $50 for a 200 mg vial [22]. Figure 1 correspondingly illustrates how the percentage of nano-vaccines out of the total number of COVID-19 vaccines procured during 2020 was significantly lesser in the middle-income countries than in the high-income ones, with the purchases of the poor countries being so low as to not even make it to the statistic, raising general questions over the availability of nanomedicines to the poor. Specifically, while three out of four COVID-19 vaccines procured by the rich countries by the end of 2020 were nano-vaccines, only one in ten vaccines were nano-vaccines in the procured stocks of the poorer countries (Figure 1b), indicating that the wealth and the privileged status coming with it are a significant determinant of the people's access to immunization against a major health treat that SARS-CoV-2 virus represents. Further, the concerns that the scenario of H1N1 vaccine hoarding by the affluent countries [23] may repeat are indicated by the disparity between the procurement of the total number of COVID-19 vaccines per capita by the rich and the poor countries. Thus, for example, compared with Brazil (GDP per capita of $6450 as per IMF 2020 statistics), which has reserved a little less than one vaccine dose per capita by the end of 2020, and Venezuela (GDP per capita of $1739), which has ordered one dose for three people, Canada (GDP per capita of $42,080) has purchased 9 vaccine doses for each person [21]. In fact, the World Health Organization (WHO) announced that by 17 January 2021, 39 million COVID-19 vaccine doses were administered in wealthy countries, as opposed to only 25 doses across all poor countries [24]. The same trend has applied to total nano-vaccine purchases per capita by 19 January 19, 2021, which follow an almost linear dependence on the nominal GDP per capita of the countries (Figure 2A). On average, high-income countries have procured a little over 1.5 nano-vaccine doses for each of their citizens, while middle-income countries have bought a little less than 0.1 nano-vaccine doses per capita (Figure 2B) and the low-income countries once again did not even make it to the statistic because their purchases have been negligible. Another way by which the rich countries have engaged in the race to ensure sufficient vaccine immunization for their citizens was by diversifying the vaccine types procured. Thus, for example, EU, UK and Canada have obtained seven different vaccine types each, while the whole African Union has obtained only three. Once again, the same trend applies to nano-vaccines, with the rich countries having reserved just over two different nano-vaccine types on average, as opposed to middle-income countries, for which the average number of different nano-vaccine types reserved equals 0.68 (Figure 2c). Overall, these findings demonstrate that COVID-19 vaccine distribution, at least in the early stages of the process, has been determined more by the wealth and the status than by the need.

**Figure 3.**
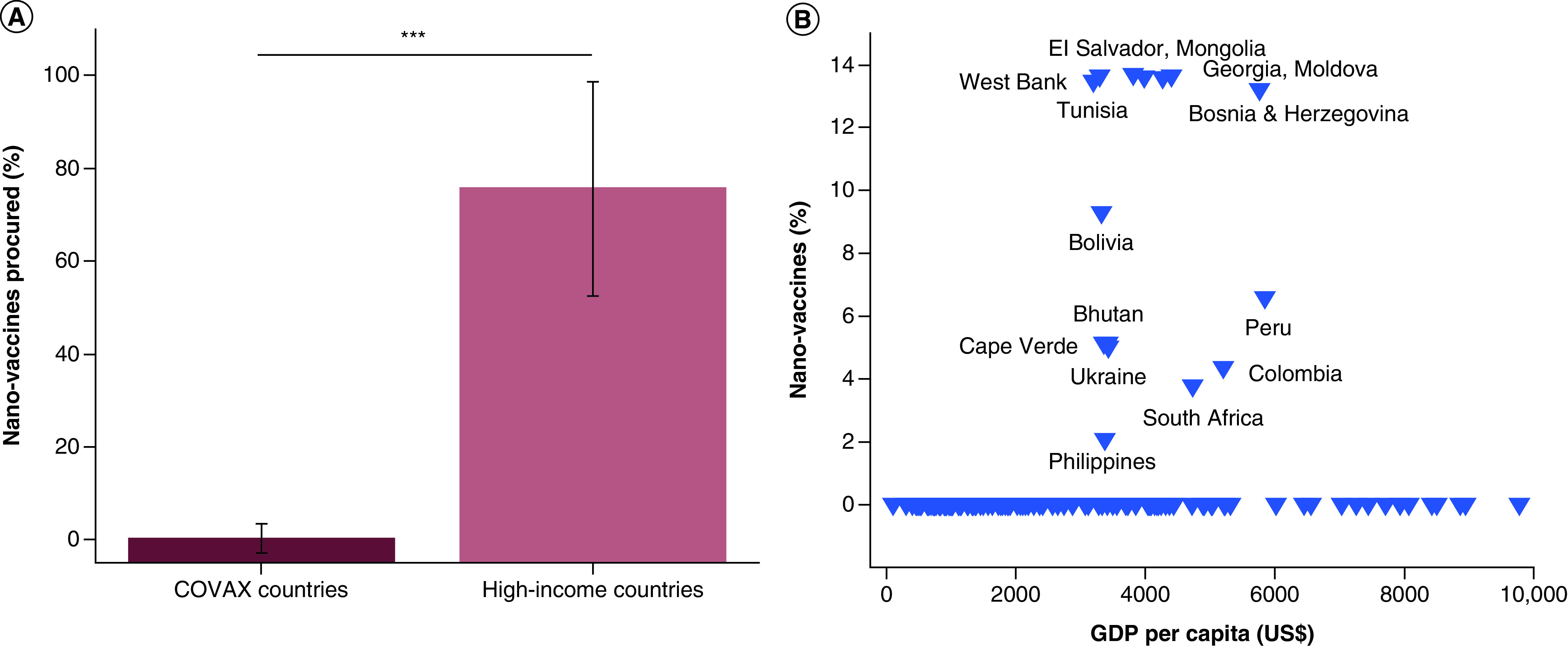
COVID-19 nano-vaccine procurement through the COVAX initiative. Average percentage of COVID-19 nano-vaccines secured by middle- and low-income countries through the COVAX initiative compared with that procured by only the high-income countries by the end of 2020 **(A).** Percentage of COVID-19 nano-vaccines secured by 118 middle- and low-income countries through the COVAX initiative by 3 February 2021 as a function of their nominal GDP per capita, as reported by the International Monetary Fund for 2020 **(B).** Data points were collected from the Duke Global Health Innovation Center COVID-19 Launch and Scale Speedometer and the Interim COVAX Distribution Forecast released by the Global Alliance for Vaccines and Immunization for February 2021. GDP: Gross domestic product.

In response to these obvious disparities in vaccination accessibility, COVID-19 Vaccines Global Access (COVAX) initiative was conceived by the Coalition for Epidemic Preparedness Innovations (CEPI), Global Alliance for Vaccines and Immunizations (GAVI) and WHO to ensure a more equitable access to this primary preventive treatment. This initiative has played a pivotal role in negotiating the pooling of the resources for the development of COVID-19 vaccines, streamlining of the regulatory processes, and the waiving of the profit margins from the vaccine prices for low-income countries. However, despite the commendable effort underlying this humanitarian initiative, the success has been only partial, as it can be deduced from two main issues currently at hand: (i) insufficient doses procured, and (ii) acquisition of cheaper vaccines with lower efficacies as compared with the nano-vaccines. As for (i), 60% of the global human population, corresponding to 4.6 billion people, expects to receive the vaccine through COVAX. Considering that only 337.2 million vaccine doses have been projected for administration by the end of the first half of 2021 [26], it is quite unlikely that this would be a sufficient amount to induce the collective immunity in the poor countries of the world. As for (ii), only 1.2 million out of this first batch of 337.2 million vaccines have been nano-vaccines, specifically the *Pfizer-BioNTech* one, the rest being the more traditional, non-replicating viral vector based one marketed by *AstraZeneca*. With only 0.35% of procured vaccines being nano-vaccines, this percentage is by almost two orders of magnitude lower than the previously derived 10.57% of nano-vaccines relative to the total vaccines for COVID-19 procured by the middle-income countries independently of the COVAX initiative (Figure 1b). It is also significantly lower than the 75.56% of nano-vaccines present in the purchased immunization lots by the world's high-income countries by the end of 2020 (Figure 3a). Due to limited supply – where the nanotechnological component of the mRNA vaccines has acted as the critical, rate-determining step in the production chain – this percentage has dropped by ∼ 20% during the month of January of 2021 [27], but it has still remained markedly higher than the 0.35% of nano-vaccines secured through the COVAX initiative.

With the proportion of failed protection against the disease being four-times higher for the *AstraZeneca* vaccine than for the *Pfizer-BioNTech* nano-vaccine (24 vs 6%, respectively), the vaccine accessible to the poor countries enrolled in the COVAX initiative is not only less expensive, but also less efficacious too, further highlighting how wealth determines the access to higher quality healthcare. In fact, medical legislatures of many rich countries have not recommended the *AstraZeneca* vaccine for people older than 65 because of its relatively low efficacy compared with the pricier nano-vaccines. This has left over 80% of this vaccine in the stocks of countries like Germany, Belgium and Italy unused and over 80% of nano-vaccines used as of late February 2021 [28]. In addition, the occasional reports of thromboembolic side effects in recipients of the affordable *AstraZeneca* vaccine have indicated that the problems with it may extend from the domain of efficacy into the domain of safety, explaining why some wealthy countries currently consider withdrawing this vaccine from their immunization campaigns. If some scientists see this as a triumph for nanomedicine, it is a bittersweet one considering the unfair, wealth-dependent patterns of distribution of these nano-products. For, not surprisingly, the *AstraZeneca* vaccine, which people in rich countries have been reluctant to receive, currently presents over 99.5% of the lot accessible to countries enrolled in the COVAX initiative. Further, on 24 February 2021, Ghana became the first out of 165 countries enrolled in COVAX to vaccinate its citizens through this global initiative, by which date vaccination campaigns had been well under way in the wealthy countries, going on for over 2 months, i.e., since mid-December 2020.

As it can be seen from Figure 3b, however, no direct correlation between the GDP per capita of countries that obtained COVID-19 vaccines through the COVAX initiative and the percentage of nano-vaccines in their procured lots can be established, except for the congregation of all the countries with nano-vaccine acquisitions in the middle-income range of $3000–$6000. On the positive side, this speaks in favor of a fairer distribution mechanism ensured through COVAX, with the total number of doses supplied to each country being proportional to its population size. On the negative side, however, no country with the GDP per capita lower than that of the Philippines ($3373 as per IMF 2020 statistics), which includes the world's 70 poorest countries and all the low-income countries, has received a single nano-vaccine dose through COVAX. African Union has signed up for the *Pfizer-BioNTech* nano-vaccine in the amount of 7.5% of the total [27], but as with the COVAX chain of supply, the allocation of nano-vaccines is likely to be based on criteria that include the readiness of the healthcare system to accept them [29], which will exclude the low-income countries as candidates. Although more demanding, low-temperature storage conditions may be one of the obstacles before the acquisition of some of the nano-vaccines, such as the *Pfizer-BioNTech* one, by the world's poorest countries, their high cost and the competition for market shares and revenues represent more important factors that have led to this situation. Still, in spite of the limited success, initiatives such as COVAX should be commended for their fostering global networks of cooperation in lieu of competition and attempting to heal the chasm of social inequity through equitable distribution of COVID-19 vaccines. Such initiatives should be expanded through greater levels of international cooperation and social activism to ensure a higher level of success compared with the rather rudimentary one achieved so far.

**Figure 4.**
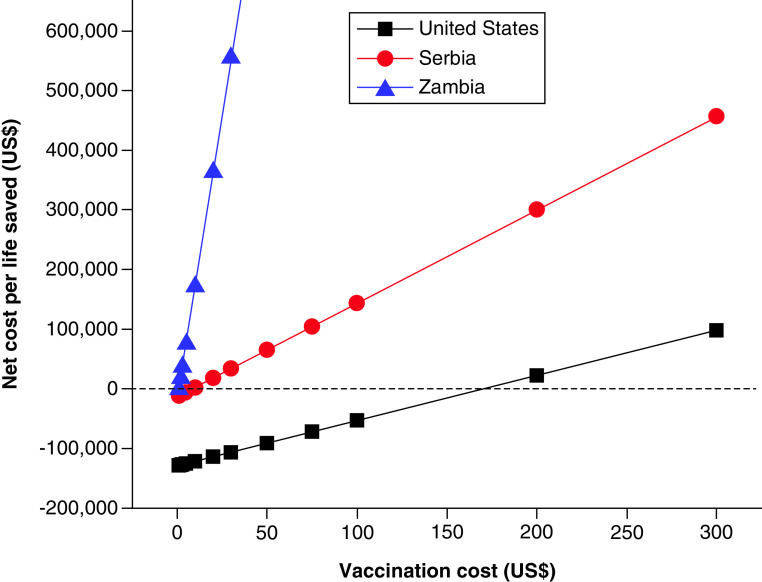
Price sensitivity analysis of saving lives with COVID-19 vaccines. Price sensitivity plots for the net costs of saving lives with vaccination in USA as a high-income country of choice, Serbia as a middle-income country of choice and Zambia as a low-income country of choice. With nominal dross domestic products per capita of $63,051, $8506 and $1001 for USA, Serbia and Zambia, respectively, the ratio between these successive values is consistently in the 7.5–8.5 range. Note the major effect of the vaccine cost in the healthcare system of a low-income country and the lesser effects in the healthcare systems of middle- and high-income countries.

Another analysis pertaining to COVID-19 nano-vaccines has been undertaken to further accentuate how the development and utilization of expensive nanotechnologies benefit the rich more than the poor and deepen the gap between the two. Specifically, Table 1 presents the calculated average net cost per life saved and the net cost per quality adjusted life year (QALY) saved by vaccination with the relatively pricey COVID-19 nano-vaccines. In this analysis, the median cost of COVID-19 treatment in USA of $3045 [30] and the 20 million diagnosed cases in 2020 were used to create one estimate of the annual burden of the disease to the healthcare system of this high-income country, equaling $60 billion. Another estimate came from 700,000 hospitalizations due to COVID-19 in 2020 [31], at the median cost of $40,000 [32], yielding $28 billion. Then the same proportion of direct medical costs due to COVID-19 compared with total annual health expenditures was applied to two more countries included in the analysis: Serbia as a middle-income country of choice and Zambia as a low-income one. The results show a stunning difference in terms of the gain or cost to a healthcare system with every life saved due to vaccination with nano-vaccines. Namely, while the healthcare system in USA gains $90,800 on average with every life saved, it costs Serbia $65,600 to save a life with vaccination. In Zambia, even more critically, the cost of saving a life exceeds $1 million. The analysis of the sensitivity plots in Figure 4, in fact, shows that the cost of a two-dose nano-vaccine would need to be lower than or equal to $8 in Serbia and lower than or equal to $1 in Zambia for saving lives in these healthcare systems to produce an economic gain. In contrast, it would take more than $170 for the price of the two-dose nano-vaccine to turn gains into costs in the massive, high-cost healthcare system in USA, slightly less sensitive to pricing than the healthcare system of Serbia and significantly less sensitive than that of Zambia (Figure 4). The net healthcare costs per life year saved and per QALY follow the same trend as that observed for the nest costs per life saved with COVID-19 nano-vaccines, being negative in USA and positive in Serbia and Zambia (Table 1). Altogether, these results demonstrate that a technology that could be applied with economic gains in an affluent social setting often creates unbearably high costs when applied in the very same form in a poorer setting. Economically feasible innovation in a high-cost healthcare system, in other words, does not translate directly to a similar feasibility in lower-cost systems. These sole economic considerations add to the overall difficulty with which advanced nanotechnologies - such as the mRNA delivery with the use of lipid nanoparticles intrinsic to the majority of first-generation COVID-19 nano-vaccines – can be reproduced in less developed settings, which are often deprived of the capacities for the synthesis of equivalent formulations. This shortage in scientific expertise and technological underdevelopment often result in the dependence on foreign trade, which further adds to the cost of the therapies and exacerbates social inequalities.

**Table 1.** Cost-effectiveness analysis for the net costs of saving lives with COVID-19 nano-vaccines in USA as a high-income country of choice, Serbia as a middle-income country of choice, and Zambia as a low-income country of choice.

Variable	Base case point estimate for USA	Base case point estimate for Serbia	Base case point estimate for Zambia	Explanatory notes
A. Nominal GDP per capita	$63,051	$8506	$1001	
B. Nominal GNI per capita	$65,760	$7020	$1440	
C. Population	328.2 million	6.9 million	17.8 million	
D. Population eligible for vaccination	246 million	5.2 million	13.3 million	75% of the total population exceeds 16 years of age
E. Annual healthcare expenditures per capita	$11,580	$530	$68	
F. Projected direct healthcare cost due to COVID-19 in 2020	$44 billion	$44 million	$14 million	Average of two independent estimates for USA (see discussion)
G. Projected direct healthcare cost relative to annual healthcare expenditures	1.1%	1.1%	1.1%	Deduced for Serbia and Zambia based on data available for USA. Note the ability of 1.1% to drastically destabilize the healthcare systems and even more so the overall economies
H. Expected mortality reduction with vaccination	95%	95%	95%	
I. Average life years gained with vaccination per patient	15.2	15.2	15.2	I = H x 0.16
J. Direct healthcare cost recovered with COVID-19 vaccine per annum	$41.8 billion	$41.8 million	$13.3 million	J = F x H
K. Health utility score of a year of life gained through averted illness with vaccination	1.0	1.0	1.0	
L. Deaths in 2020 due to COVID-19	342,000	3500	730	
M. Number of lives saved per year with vaccination	324,900	3325	693	M = L x H
N. Net cost per life saved with nano-vaccines	-$90,800	$65,620	$1,265,000	Nano-vaccine therapy estimated at $50 on average for a double dose per person. N = [($50 x D) – J]/M
O. Net cost per life year saved with nano-vaccines	-$5970	$4320	$83,230	O = N/I
P. Net cost per QALY saved with nano-vaccines	-$5970	$4320	$83,230	P = O x K

## Nanomedicine versus social inequalities: top-down perspective

Previous decades have witnessed a rise in the commercialization of biomedical science, a trend that nanomedicine has not been immune to by any means. Aside from the favoring of applicative research over its fundamental equivalent and the fosterage of the optimization of ongoing technologies more than the search for the radically new concepts, this corporatization of medical research even in its most free-minded, academic settings has triggered another major pandemic, namely that of historical and sociological illiteracy. By narrowing down the scope of scientific thought only to those channels where the flow of cash could be promoted, commercialization of academic science has had a devastating effect on the scientists' awareness of the effects that science has on the society. Half a century ago, it was customary for scientists to engage in the critical discussion of positive, but also potentially detrimental social effects of their research, such as the destructive power of the nuclear energy in the atomic age. Today, however, one such essentially moral discourse has been largely washed away by this cash flow, even though technologies that include genome editing tools, e.g., CRISPR/Cas9 or TALEN, organ-targeting nanoparticles or remotely accessible implantable diagnostic devices, now pose equal threats to an irreversible alteration of the fabric of human life as the nuclear energy did at its prime. However, commercialization has turned science into a new religion, with ever so little critical thought and responsibility with regard to how science affects the local and the global communities.

Correspondingly, the gap in scientific and technological competence between the rich and the poor stems largely from the lack of understanding of the extent to which socioeconomic factors and science affect one another. It is common, for example, for the affiliates of the R&D centers in the developed world to denote the weak economic prospects of poor countries and their meritocratic incapacity as the main reasons why advanced technologies can seldom be applied in their settings. However, it is often ignored that underdevelopment is usually caused by political, geostrategic and other endemic factors that are beyond the sphere of influence of ordinary people. A related oft-repeated argument is that technologies must mature before their translation to poorer settings may occur, even though not all technologies mature, not even in the affluent venues. One example comes from growth factors, which were once considered as most prospective ingredients of tissue engineering constructs. Their main weakness, however, turned out not to be their exorbitant cost, which often exceeds $10 million per gram as for bone morphogenetic proteins or even $100 million per gram as for TGF-β1, but rather the tendency to produce abnormal tissue growth in lieu of healing [33]. And yet, for the unsafe use of growth factors to be recognized, the scientific milieu had to pass first through the phase of hype over these chemicals once hailed as magic healers in tissue engineering [34], then through the stage where disappointing clinical results began to accumulate [35], and, finally, it had to witness a moment when a major scandal involving a world's largest medical device company, *Medtronic*, was revealed, bearing a heavy toll on patients [36]. This, of course, does not mean that a once-failed technology can never become a success; rather, most likely, the growth factors have not lived up to the ambitions of their pioneers because their potent activities require next-generation drug carriers capable of achieving more precise spatiotemporal deliveries of therapeutic cargos than it is possible today. However, the failure of one technology paves way for the stream of success of another and their resurrections are, for this reason, very difficult to achieve in reality. Still, with poor countries often becoming graveyards for old technologies, revitalizing their potential for use can be counted among the research approaches inherently favoring the closing of the gap between the rich and the poor.

Nevertheless, in many cases, the potential of a new technology is unequivocally praised at first, the reason being the desire to collect funding for its R&D, and only then, years down the road, suspicions over it become aired, coinciding with the dried pipeline for then not anymore so trendy of a topic. Such was the case with the enhanced permeability and retention (EPR) effect, a long-standing paradigm in the field of nanomedicine [37]. Although the effect sounded too general to be true even early on, the concerns over its implausibility began to be exposed only after the first wave of excitement over it had passed, having filled many a coffer by then. Now that the disparity between the large number of preclinical papers with positive findings and no corresponding products reaching the market has raised serious questions over its validity [38], we know that the effect is far more complex that it was initially thought. Numerous microanatomical and biochemical factors are now known to affect the extravasation of nanoparticles through pores in the endothelium of a leaky vasculature within and around the malign tissue [39], making the effect pronouncedly patient- and tumor-dependent [40]. However, only when EPR as a topic became partially trite and the frustrations over the lack of funding started piling up did the reports begin to appear that put this effect in a more critical light, showing us how money, unfortunately, can have a decisive say on how prospective, if not true, a certain model is considered by a scientific society. And it is always frightening to realize the extent to which we, who were to be the users of a tool, have become its own inert tool. Some of the major decisions we make in our creative work as scientists are subdued to the money's interest to grow more money - an insight that is nothing short of disheartening.

But there is hope and it lies in drifting away from this corporate mentality and liberating the scientific thought process from the dependency on fiscal matters and everything tied to them. This would be a step toward endowing the poor with a voice and have them no longer be passive observers of the money-centered game of innovation for the privileged. At the same time, stepping out of this game that favors the big and swallows the small may come up with a lot of surprising benefits. For, small and intimate R&D settings may be those where innovative thought thrives most prolifically, even though they are invariably short of resources. In art and in many other spheres of human interest, in fact, transformative new ideas arise most profusely from smallness and destitution [41]. However, in science, for some reason, the dominant paradigm has been that commercial interest, privatization and intellectual property (IP) protection are the drivers of innovation, simply because no alternative models have been put to test in practice. IP enforces industrialization, the death sentence to any art and humanity, and yet this model lingers like an incontestable creed in the province of science. It favors the inflation of research venues into robust bureaucracies, which are at fundamental odds with the ‘wayward’ thinking regimens leading to groundbreaking discoveries. Its expectance of large-scale commercialization stands in the way of the fosterage of many of the authentic lo-tech, slow-tech, easy-tech and retro-tech alternatives to the high-tech philosophy prevalent in the medical niches of the western world. IP, moreover, perpetuates the capitalist economy of science, where knowledge takes the form of an asset for the accumulation of financial capital, favoring marketable solutions that bring a short-term profit over the discovery or invention of fundamental new concepts, which may take longer than the inventor's lifetime to ripen into practical applications. On the back of the increasing failure of drugs in the late stages of development, IP has also acted as a source for the ongoing epidemic of drug repurposing, which does not only surrender the rational design capabilities of pharmaceutical science, but also reiterates how huge of financial obstacles stand in the way of developing new drugs, thus reinstating the fact that pharmaceutical science is, before all, founded on financial interests rather than on sound science or transboundary humanitarianism. Ironically, with its focus on personal profit and protection, IP depersonalizes the R&D sector and overshadows its inherent altruism, contributing to the epidemic of toxic workplaces that are all but conducive to harmonious medical research and practice. Its clandestine nature feeding on the newly installed data exclusivity is also in conflict with the increasingly open world of science [42], where free sharing of ideas facilitates progress and builds altruism around which the most creative medical research can flourish. And yet, as exemplified by the case of Indonesia's withdrawing from the Global Influenza Surveillance Network (GISN) agreement of sharing the viral samples with the WHO during the H5N1 avian flu epidemic after it was found out that the donated samples were used by an Australian company to develop a flu vaccine available to Indonesia for commercial purchase only [43,44], the entanglement of the ideology of free sharing within the network of IP and private property can elevate the levels of global mistrust and create further indentations in the already wide gap of social inequity.

It goes without saying that, morally speaking, it is erroneous to prioritize profits over the wide accessibility and affordability of medicines, as it is the case in many developed countries [45]. That civil rights should come before property rights is evident, but it is equally evident that basing scientific and technological progress on property rights takes away the rights to universal healthcare and can be questioned for its dubious ethicality. In reverse, to provide access to medicines as a basic human right, just as well to see all medical technologies as such, may not be possible in a current system where scientific innovation is enabled on the basis of profit-seeking objectives. Instead we have a global situation where despite the flexible rights to grant compulsory licenses and other exemptions of the Doha Declaration, the Agreement on Trade-Related Aspects of IP Rights (TRIPS) by the World Trade Organization strongly enforces the IP protection to monopolize the market, limit the access of the poor to affordable medicines and maximize profits at the cost of deteriorating public health, not only in the developing countries anymore, but also in the high-income ones [46,47]. Even the relaxed measures of the TRIPS agreement cannot be taken advantage of in an emergency situation such as the ongoing pandemic because of the insufficient resources, infrastructure and know-how to recreate a COVID-19 vaccine in an underprivileged setting. Simultaneously, private corporations acquire the IP rights on publicly funded research, be it by directly receiving the federal funding or by exploiting the equitable licensing policies in academia, thus forcing the patients to pay both for innovation through taxes and for the pharmaceutical or medical products in need of [48].

And yet, innovation in medical nanotechnologies could be created and delivered freely to the people. One example may be the recent design and fabrication of SARS-CoV-2 protective garments containing copper oxide nanoparticles and graphene nanosheets as inorganic antivirals without any IP protection and with openness to freely share the method of production and assist in its translation to different settings [49]. After all, given that the recent push to waive the IP protection rights for COVID-19 vaccines so as to make them available to developing countries through local production and thus ameliorate the aforementioned issue of their wealth-dependent distribution was met with firm resistance from the pharmaceutical industry [50], the only path open before many socially aware, anti-elitist, people's innovators is that of engaging in free sharing of ideas and products, against the premises of the neoliberal market ideology. These little acts of resistance against the dominant economic model, where the soaring costs of medicines obstruct the access to affordable medical products, strain the health budgets and deteriorate the quality of healthcare, is how grassroots incentives for renewing the current system are being disseminated. In times of crisis, like this, they provide an opportunity to set a landslide of concordant sentiments in motion so as to wash away the features of an inherently unfair system and set the foundations for the installment of something fairer and more humane in its place.

The case of artesunate and amodiaquine, two therapeutic molecules invented, developed and clinically tested in China during the period of public property can be instructive here [51]. The fact that neither of these two small molecules were being patented allowed the humanitarian organizations, *Médecins Sans Frontières* (MSF) and the Drugs for Neglected Diseases initiative (DNDi) to utilize them in an antimalarial formulation, which would be later sold by *Sinofi* in the no-profit value regime, with the price formulas adjusted to the production cost. Rather than maximizing the financial returns at the cost of diminished accessibility and affordability and destabilizing the social payers via capital inflation, this selling of a medical product without a patent or exclusionary license and without any profit-seeking intentions attracted a cooperative network of academic research labs, public consortia and humanitarian organizations. The fact that the great majority of these academic labs, university spin-offs and biotech startups provided their know-how or technologies free of charge, as a common good, without receiving any IP rights in return, helped to break down the barrier of private ownership and make the antimalarial drugs more available to the low-income population. Even at the cost of $1 per course for adults and ¢50 for children, however, this combination therapy continues to be unaffordable for the majority of the affected population [52], which suggests that ever more radical measures stemming from the science and economics of frugality and egalitarianism must be taken before innovative medical technologies can become fully accessible to the poor.

This brings us over to the second way by which nanomedical research can contribute to healing the gap between the rich and the poor, complementing the previously mentioned choice over the research subjects. It involves control over various organizational aspects of research and their adjustment in the direction of challenging the commercial, competitive realities in which scientific innovation of the day operates. If successfully conducted, rewards along this line of effort will come not in the form of monetary satisfaction, but rather as a contribution to social welfare and harmony, as inconspicuous as this can be. In short, it takes countering the capital, giving away the rewards and siding with the poor to heal the system for real. Or, as medical practitioners often say, it must get worse before it betters, darker before it brightens. On the way to these horizons, it is wise to find solace in what Jonas Salk, the inventor of the first approved polio vaccine and a scientist once described as “the foster parent of children around the world with no thought of the money he could make by withholding the vaccine from the children of the poor” [53], said when asked who owned the patent for the vaccine: “Well, people, I would say. There is no patent. Could you patent the Sun” [54]?

No doubt, for research in nanomedicine, like that in any other field of science, to contribute to healing the social inequality divide, the tensions between corporate profit and public health, which have rendered the system broken at so many places, must be resolved. Alas, for these resolutions to be reached and the sun of science of the people, by the people and for the people liberated, many scientists will need to turn into politicians, if not peaceful revolutionaries, before they would find the return to the escapades of the lab life morally justified.

## Conclusion

Scrutinizing scientific research, including that falling under the umbrella of nanomedicine, from various historical, sociological and philosophical angles is of vital importance for ensuring the progress of science in socially benevolent directions. The concern that nanomedicine is becoming an exquisite technology for the privileged has been discussed here. This concern was backed by showing a dramatic wealth-dependent disparity between the access of different countries to nano-vaccines, which have emerged as both more expensive and more effective than their traditional counterparts. Specifically, while three out of four COVID-19 vaccines procured by the rich countries by the end of 2020 were nano-vaccines, one in ten vaccines were nano-vaccines in the procured stocks of the middle-income countries. Even more critically, only one in 285 vaccines in the modest stock secured by the COVAX initiative for immunization of people in the world's poorest countries throughout the first half of 2021 were nano-vaccines. This trend has demonstrated a direct correspondence between the wealth of a country and its access to advanced, nanomedical care.

Lest this disparity continue to deepen the global gap of social inequality and create countless adversities in its wake, it must be addressed in a timely manner. To understand where the incentives for amending this disparity should be directed to, it pays off to understand what has caused it in the first place. Using this approach, two routes leading to the amelioration of this trend have been derived: bottom-up and top-down. The former, bottom-up route stems from the fact that no technologies are neutral, as each of them are predisposed for a specific socioeconomic impact, the analyses of which should be performed in early stages of the conception of these technologies. Such analyses are thoroughly uncommon, but are a necessary accompaniment of the development of socially responsible technologies. However, for their implementation to become more pervasive among the intellectual forces developing the new technologies, the dominant economic models governing this process, limiting themselves to monetary considerations only, must be shunned in favor of broader models, which would take into account the ecological, cultural and humanistic dimensions of their application. If we accept that the goal is not only the financial only, namely to increase the wealth of the poor, but also such that it has all these additional dimensions ascribed to it, we would be brought to the doorstep of more holistic models for assessing the socioeconomic footprint of medical nanotechnologies.

This brings us over to the second, top-bottom route necessary to follow to ensure that nanomedicine contributes to the state of social harmony instead of tearing the social fabric along the cracks separating the rich from the poor. It is that of social activism directed at amending the aforementioned models underlying the discovery and development of new technologies. The premises of competition-driven innovation and other aspects of the ideology of neoliberal capitalism may be effective in terms of outcomes, but the means leading to these outcomes produce arrays of irremediable side effects, one of which has been the continuous deterioration of social and healthcare equity. If nanomedicine is to deliver its promises to the people independently of the class, caste or color rather than be a resource for the handful of the privileged, drastic changes are to be introduced, both in the way we view the fundamentals of scientific research and innovation and in the way the web of socioeconomic relations connecting the bench to the bedside is being spun and sustained.

## Future perspective

When trying to get a glimpse of the most prolific future directions for subjects at hand, it often pays off to return to the earliest beginnings. In this case, this starting point can be the question present in the title of this paper, itself an embodiment of wonder over the effects nanomedicine has and will have on the gap between the rich and the poor. Is it impossible for the critical mass of nanomedical researchers to think broadly enough to bring about a fundamental change in the way innovations in this field are being discovered and delivered to the people? Or, perhaps, with the proper incentives, the change is not unthinkable and all that is needed is well-timed outreach, which this perspective article has aimed to provide. If it is so, then there is a lot of work ahead of us because what is needed is to overhaul the fundamentals of not only the frameworks of research and discovery, but also of the reigning economic and political models through the application of which nanomedical inventions become available to the people. Common to both of these reformations is humanization of the sociopolitical structures in place and the contents that they churn out. This humanization is a direct outcome of no longer considering purely scientific matters in the innovation stage and purely economic matters in the application stage, but rather extending these considerations to the realms of arts and humanities. Time will show that neither the scientific rigor would be diluted nor the economic relations rendered less efficacious with this expansion of both into more holistic territories. Rather, there is a reasonable expectation that both science and economy could be enriched and the flourishment of social harmony secured if routes for their humanization outlined here shall have been pursued.

**Executive summary**

**Nanomedicine versus social inequalities**

- The income inequality directly leads to social inequity in unmet healthcare needs and it is discussed how nanomedicine could contribute to shrinking this gap between the rich and the poor instead of becoming an exquisite technology for the privileged.

**Nanomedicine versus social inequalities: bottom-up perspective**

- There are no neutral technologies; innovation in medical nanotechnology intrinsically shapes human values and modes of communication.
- The birth and the fosterage of a new technology can change the socioeconomic aspects of the social system in which it is applied.
- The more frequent use of contextual analyses to derive research topics that positively affect the sociocultural climate emerges as a route through which nanomedical research can reduce the social inequality divide.
- These qualitative analyses go beyond mere considerations of scientific subjects and foster the inclusion of views from various humanistic angles.

**Example of COVID-19 nano-vaccines**

- COVID-19 vaccines incorporating nanotechnology are more efficacious, but also more expensive than the more traditional vaccines.
- Nanotechnologies incorporated as delivery mechanisms for immunostimulatory mRNA in COVID-19 nano-vaccines are the main contributor to their comparatively high costs.
- COVID-19 vaccine distribution at the international level has prioritized the wealth and the status of the countries over people's needs.
- Total nano-vaccine purchases per capita and their proportion within the total vaccine lots follow an almost linear dependence on the nominal GDP per capita of the countries.
- By January 2021, high-income countries procured over 1.5 nano-vaccine doses per capita, while middle-income countries procured less than 0.1 nano-vaccine doses per capita.
- While three out of four COVID-19 vaccines procured by the rich countries by the end of 2020 were nano-vaccines, only one in ten vaccines were nano-vaccines in the procured stocks of the middle-income countries and only one in 285 vaccines were nano-vaccines in the stocks secured by the COVAX initiative for the world's middle- and low-income countries.
- No country with the nominal GDP per capita lower than $3,370, which includes the world's 70 poorest countries and all the low-income countries, has been projected to receive a single nano-vaccine dose before the end of the second quarter of 2021.
- Cost-effectiveness analysis has shown that nano-vaccines produce a massive net healthcare gain for saving lives in USA, but because of their high cost, they produce net healthcare costs for saving lives in middle- and low-income countries.
- COVID-19 nano-vaccines exemplify a medical nanotechnology applicable with economic gains in an affluent healthcare setting, but with high costs in poorer settings.
- Currently, the development and utilization of expensive nanotechnologies benefits the rich more than the poor and deepens the gap between the two.

**Nanomedicine versus social inequalities: top-down perspective**

- Commercialization and corporatization of science tends to wipe out the critical thought and has limited the scientists' awareness of the effects science has had on the society.
- The gap in scientific and technological competence between the rich and the poor stems largely from the lack of understanding of the extent to which socioeconomic factors and science affect one another.
- Numerous benefits may result from the liberation of the scientific ideas and work plans from their tendency to inertly follow the monetary streams.
- Innovation in medical nanotechnologies could be created and delivered freely to the people, without hindrances in the form of intellectual property or other exclusionary rights and regulations.
- Control over various organizational aspects of R&D and their adjustment in the direction of challenging the commercial and competitive grounds for scientific innovation emerges as another route through which nanomedicine could heal the social inequality divide.
